# Prevalence of classic and non-classic pain sites of coronary artery disease: a cross-sectional study

**DOI:** 10.1186/s12872-024-04127-z

**Published:** 2024-08-24

**Authors:** Mayar Abdullatef, Maya Omran, Anas Bitar, Bayan Alsaid

**Affiliations:** 1 Faculty of Medicine, Damascus University, Damascus, Syria; 2 Laboratory of Anatomy, Faculty of Medicine, Damascus University, Damascus, Syria

**Keywords:** Coronary artery disease “CAD”, Classic, Non-classic, Ischemic heart disease, Syria

## Abstract

**Study objective:**

This study aims to assess the prevalence of both classic and non-classic pain sites in patients with ischemic heart disease, emphasizing the importance of recognizing and not disregarding non-classic symptoms.

**Methods:**

This cross-sectional study included 100 patients diagnosed with coronary artery disease (CAD) who were admitted to two major hospitals in Syria. classic pain was identified as pain located in the precordial area, with or without radiation to the neck, jaw, left shoulder or arm. Patients’ demographics and previous medical history were documented to investigate any potential associations with non-classic pain.

**Results:**

62% of the patients experienced non-classic pain, while 12% had no precordial pain. For those without precordial pain, the most common pain site was the left chest (66.7%). Non-classic pain was significantly associated with smoking, with 72.2% of smokers experiencing non-classic pain compared to 35.7% non-smokers (*p* = 0.001). Additionally, patients with previous heart disease were more likely to have non-classic pain (71.7%), compared with patients with no history of heart disease (51.1%) (*p* = 0.03). Other factors such as age, sex, and diabetes were not statistically significant.

**Conclusion:**

Non-classic pain is common, affecting 62% of individuals, primarily in the right shoulder, right arm, and back. This type of pain could be associated with smoking and prior heart disease. Misdiagnosing coronary artery disease can have serious consequences, as patients with non-classic symptoms may miss important pre-hospital procedures like ECG.

**Supplementary Information:**

The online version contains supplementary material available at 10.1186/s12872-024-04127-z.

## Introduction

Coronary artery disease (CAD) is one of the major cardiovascular diseases affecting the global human population [1]. Environmental factors, genetic factors, unhealthy lifestyle, chronic diseases and many other factors could cause CAD, culminating in cardiac ischemia and ultimately progressing to myocardial infarction [2].

Pain induced by CAD is called angina pectoris, and it is commonly characterized by a slow onset of retrosternal chest discomfort, which can be brought on by physical or emotional stress and may even occur at rest in cases of acute coronary syndrome. The discomfort may spread to the left arm, neck, or jaw, teeth, and, ear [3]. This radiation may be caused by the convergence of the vagus, trigeminal and cervical spinal nerves (C2-C3) [4, 5]. In addition, the discomfort is often accompanied by symptoms such as difficulty breathing, nausea, and dizziness [6]. A study found that the majority of female and male participants exhibited symptoms of chest pain, and this type of pain is more common than atypical symptoms [6, 7]. Atypical pain is frequently defined as epigastric or back pain or pain that is described as burning, stabbing, or characteristic of indigestion [8].

Angina pectoris has two types: stable angina and unstable angina. Unlike stable angina, which is usually induced by exertion, unstable angina presents sudden symptoms even while at rest [9]. Patients with unstable angina have a worse prognosis and are more likely to develop MI [9]. Patients with CAD may experience classic/typical or non-classic/atypical pain. Classic pain site presentation has been defined as having midsternal or having midsternal with radiating left neck, shoulder or arm pain/discomfort of any or at least moderate intensity [10]. A previous study revealed that in certain cases, craniofacial pain was the only complaint during the ischemic episode [5].

Patients with atypical pain are less likely to receive a diagnosis and consequently have a mortality rate three times higher than patients with typical/classic angina symptoms [5]. Furthermore, a significant proportion of myocardial infarctions (MIs) are asymptomatic or present with minor and non-classic symptoms, and are incidentally detected during routine electrocardiogram (ECG) screenings that show the presence of abnormal Q waves [11]. Nonetheless, the patient may present with heterotopic pain (pain occurring in a region despite the real source being elsewhere in the body) in this region, with the real source potentially being of cardiac origin [12]. The cardiac heterotopic pain can lead to misdiagnosis and unnecessary medical procedures as was shown in several reports [13].

Several patients with CAD present with non-classic symptoms that are not detected in the emergency department using the standard diagnostic methods of history taking, physical examination, and 12-lead ECG. If these patients are not hospitalized for additional assessment, the diagnosis may be missed. The 2–5% of MI patients who are inadvertently discharged home frequently have poor outcomes, making them a significant source of malpractice claims in emergency medicine [14]. Misdiagnosed cases could develop lethal complications. Absence of chest pain and the lack of elevation of ST segment in electrocardiogram “ECG” were the main causes of misdiagnosis, as was shown in a previous study [15].

It is important to note that the term “Atypical pain” is a misleading way to describe chest pain, and its use is not recommended [6]. In our study, we opted for the terms “classic” and “non-classic” to describe different types of pain, as these terms provide a more accurate representation of the symptoms experienced.

To our knowledge, this is the first paper to study the prevalence of classic and non-classic pain sites of cardiac origin in Syria. The objective of this study was to assess the prevalence of pain sites in CAD patients, focusing on both classic and non-classic angina pain sites. In addition, we aimed to explore the factors that are associated with having non-classic pain presentation.

## Materials and methods

This cross-sectional study examined 100 consecutive patients who were admitted to two cardiology departments in two major hospitals in Damascus, Syria (Assad University Hospital and University Heart Surgery Center), and were diagnosed with coronary artery disease (CAD) by a cardiologist after having signs and/or symptoms indicating coronary artery disease according to the American College of Cardiologists’ (ACC) definition, and angiography was performed on each of the patients to determine cardiac ischemia [16]. The study period was divided into two time periods between November 2021 and December 2023, with a one-year gap in between. Exclusion criteria encompassed individuals under the age of 18, those with dental issues, psychiatric disorders, chronic headaches, or jaw masses, as they did not fulfill the study’s eligibility requirements.

**Fig. 1:**
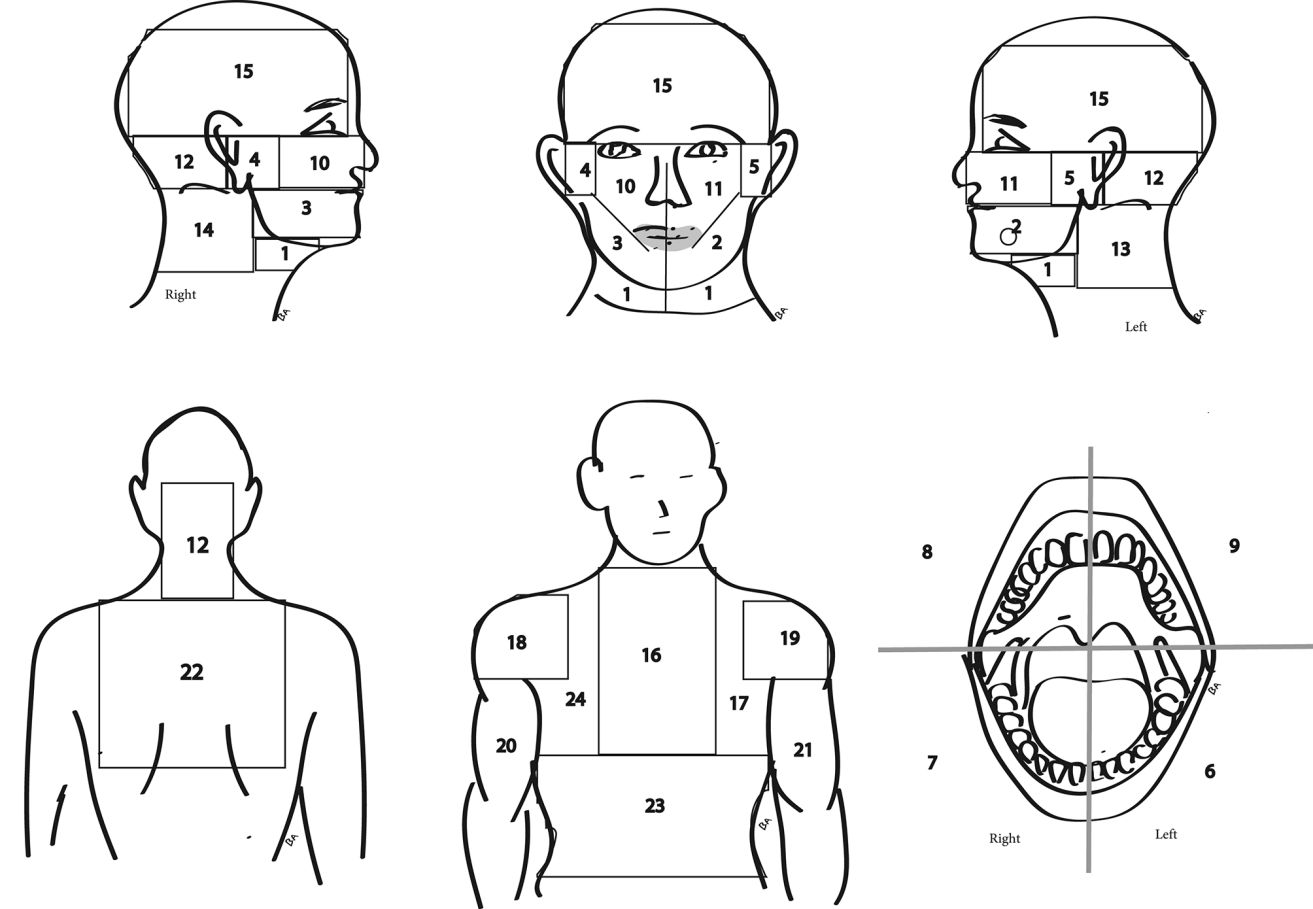
Illustration of the body and craniofacial structures divided into areas

Each patient was shown an anatomical illustration that depicts the chest, abdomen, back, shoulders, arms, face, neck and mouth [5] (Fig. 1), and was asked to identify the location of their pain or discomfort in this current situation, with the corresponding site of pain being marked. Additional required information was obtained from the patients: demographic details, risk factors (smoking, alcohol consumption, diabetes mellitus, hypertension, personal and family history of cardiac disease, and medication), personal medical and surgical history, recent dental examination and/or treatment and physical activity, and the information was recorded in data forms.

Classic pain was identified as pain located in the precordial area, with or without radiation to the neck, jaw, left shoulder or left arm [11]. Any other distribution of pain was considered non-classic. Due to discrepancies in the definition of typical/classic pain, we conducted two types of analyses: one considering the neck and jaw as possible classic pain radiation sites, and the other excluding these sites. This approach allowed us to determine if there were any differences between the two definitions.

### Ethical approval

Informed consent was obtained from each patient and the participation of the patients was voluntary.

### Consent for publication

the study protocol was approved by the Ethical Committee of Damascus University, Faculty of Medicine, Syria (document number: 4597, 25-10-2021).

### Statistical analysis

The data presentation included frequencies (%) for categorical variables and mean ± standard deviation (SD) for continuous variables. The analysis was done using IBM SPSS Statistics for Windows, version 26.0 (IBM Corp., Armonk, N.Y., USA). The Chi-Squared test was used to examine the relationship between the type of pain (classic or non-classic) and the studied variables. The age variable was dichotomized based on the median of the patients’ age (58 years). For variables where the expected frequency was less than 5 in more than 20% of the cells, the Fisher exact test was used as an alternative to the chi-squared test. The absolute Phi (Φ) factor was calculated to assess the strength of the associations. The values of ‘1’ indicate a complete association, ‘0’ indicates no association, ‘0.1 indicates a small association, ‘0.3’ indicates a medium association, and ‘0.5’ indicates a large association. Phi (Φ) was only illustrated when there was a statistical significance when using a chi-squared test. Statistical significance was set at *p* ≤ 0.05.

## Results

### Characteristics of the included sample

The study included 100 participants with a mean age of 57.88 ± 9.05. Males (79%) constituted the majority of the sample. The mean age of the male patients was 58.1 ± 8.7 years, while the mean age of the female patients was 57.1 ± 10.6 years. A significant portion of the patients (70%) lived in urban areas. Most of the patients were smokers (72%), while only a small percentage (9%) reported consuming alcohol. Diabetes was present in 38% of the patients, and more than half of them (53%) had hypertension and a history of heart disease. Additionally, 59% reported a familial history of heart disease. Detailed characteristics of the patients are presented in Table 1.

**Table 1:** Patients’ characteristics. (*n* = 100)

Variable	Frequency / Mean ± SD
**Age (yrs)** (Min: 32 - Max: 75)	57.88 ± 9.05
**Sex**	
Females	21
Males	79
**Residency type**	
Rural	29
Urban	71
**Smoking**	
No	28
Yes	72
**Alcohol consumption**	
No	91
Yes	9
**Diabetes**	
No	62
Yes	38
**Hypertension**	
No	47
Yes	53
**Previous heart disease**	
No	47
Yes	53
**Familial history of heart disease**	
No	41
Yes	59
**Recent dental procedure**	
No	85
Yes	15
**Sports**	
No	71
Yes	29

### Pain distribution

The precordial area was the most commonly reported pain site, experienced by 88% of the sample. This was followed by the left shoulder and left arm, reported by 47% and 41% of the sample, respectively. Common sites of pain also included the right shoulder (27%) and the back (24%).

Of the patients, 38% presented with classic pain, with 36.8% of the patients complaining of left shoulder pain and 36.8% experiencing left arm pain. Meanwhile, 62% of the patients presented with non-classic pain, or pain radiating to other locations such as the right shoulder, right arm, abdomen, back, or the craniofacial area. Important areas for non-classic pain patients were the right shoulder (43.5%), the back (38.7%), and the right arm (27.4%).

**Fig. 2:**
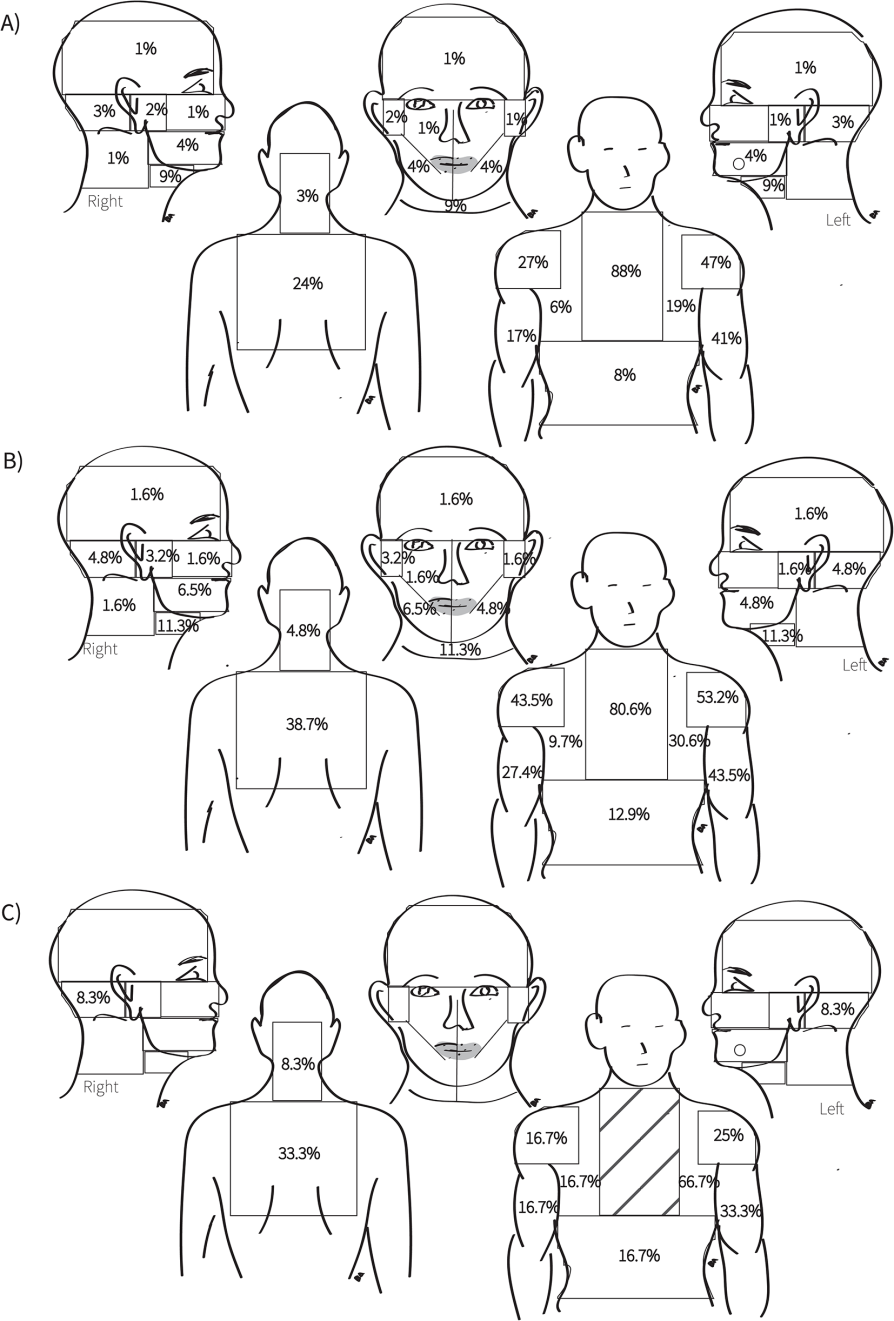
**A**. Distribution of reported pain sites (n = 100)/ **B**. Distribution of reported pain sites in non-classic patients (n = 62)/ **C**. Distribution of reported pain sites in patients with no precordial pain (n = 12)

12% of the patients presented with no precordial pain. The most common site of pain in those patients was the left chest (66.7%), followed by both the left arm and the back in 33.3% of patients. While 8.3% complained from pain in the occipital area with no chest pain. Figure 2 provides additional information on the reported pain sites, and Table 2 demonstrates the distribution of reported pain amongst smokers.

**Table 2:** Sites of pain in smokers

Site of Pain	Percentage
Precordial area Left shoulder Left arm Right shoulder Back Left chest Right arm Abdomen Right chest Neck Right mandible Occipital area Left mandible Right temporal area Left temporal area Right maxillary	83.3% 43.1% 40.3% 26.4% 25% 23.6% 19.4% 11.1% 8.4% 5.6% 4.2% 4.2% 2.8% 2.8% 1.4% 1.4%

### Variables associated with classic and non-classic pain

Smoking and previous heart disease were associated with the type of pain. Most of the patients who were smokers presented with non-classic pain (52 patients, 72.2%), compared to 20 smokers (27.84%) who presented with classic pain. While among non-smokers, 10 patients (35.7%) had non-classic pain and 18 patients ( 64.3%) had classic pain, *X*^2^ (1, *N* = 100) = 11.41, *p* = 0.001, and the association was medium (Φ = 0.34). Patients with previous heart disease were more likely to have non-classic pain (71.7%), compared with patients with no history of heart disease (51.1%) (*p* = 0.03), *X*^2^ (1, *N* = 100) = 4.50, *p* = 0.03. The association between previous heart disease and the type of pain was small (Φ = 0.21). Other variables such as age, sex, and diabetes were not significantly associated with the type of pain. Details on the variables associated with classic and non-classic pain are presented in Table 3.

**Table 3:** Variables associated with classic and non-classic pain sites presentation using Chi-Squared test^a^

Variable		Non-classic (*n* = 62)	Classic (*n* = 38)	X^2^	*p*
Age	57 and less	33 (70.2%)	14 (29.8%)	2.54	0.11
	58 and more	39 (54.7%)	24 (45.3%)		
Sex	Female	11 (52.4%)	10 (47.6%)	1.04	0.31
	Male	51 (64.6%)	28 (35.4%)		
Residency	Rural	16 (55.2%)	13 (44.8%)	0.81	0.37
	Urban	46 (64.8%)	25 (35.2%)		
Smoking	No	10 (35.7%)	18 (64.3%)	11.41	0.001 Φ = 0.34
	Yes	52 (72.2%)	20 (27.8%)		
Alcohol	No	56 (61.5%)	35 (38.5%)	-	1.0
	Yes	6 (66.7%)	3 (33.3%)		
Diabetes	No	40 (64.5%)	22 (35.5%)	0.44	0.51
	Yes	22 (57.9%)	16 (42.1%)		
Hypertension	No	32 (68.1%)	15 (31.9%)	1.39	0.24
	Yes	30 (56.6%)	23 (43.4%)		
Familial history of heart disease	No	25 (61.0%)	16 (39.0%)	0.03	0.90
	Yes	37 (62.7%)	22 (37.3%)		
Previous heart disease	No	24 (51.1%)	23 (48.9%)	4.50	0.03 Φ = 0.21
	Yes	38 (71.7%)	15 (28.3%)		
Recent dental procedure	No	56 (65.9%)	29 (34.1%)	3.63	0.06
	Yes	6 (40.0%)	9 (60.0%)		
Sports	No	43 (60.6%)	28 (39.4%)	0.21	0.64
	Yes	19 (65.5%)	10 (34.5%)		

^a^Fisher’s exact test was used where appropriate

No significant difference was observed when the jaw and neck were excluded as classic pain radiation sites, as the prevalence differed by only two patients.

## Discussion

Cardiovascular disease (CVD) is a widespread cause of socio-economic and healthcareissues globally. Morbidity, mortality, and disability caused by CVD are on the rise annually [17], accounting for 30% of all-cause mortality. Coronary heart disease (CHD) is the primary cause of death globally [18]. Patients who experience pain triggered by physical activity and relieved by rest are considered ideal candidates for cardiac pain diagnosis.

Patients with CAD may experience referred pain in different regions, such as the head, neck, arms, back, and abdominal region [13]. Several studies have explored the referred pain experienced by patients with ischemic heart disease. The objective of this study was to determine the frequency of classic and non-classic pain and to caution doctors against disregarding non-classic symptoms in hospitalized patients.

The terms typical and atypical are not consistently agreed on in the current literature, with differences in defining what defines a typical pain. However, the term “atypical” is misleading when describing chest pain and is not recommended [6]. Some of the subsequently mentioned studies utilized the terms “typical” and “atypical” anginal pain, where “typical pain” referred to discomfort felt in the chest and upper left arm, and “atypical pain” denoted discomfort in the back, neck, or jaw. In our study, we adopted the terms classic and non-classic anginal pain, and we adhered to the idea that the classic pain can be radiated to the neck and jaw, as well as to the left shoulder and arm [10]. It is noteworthy that our analysis found no significant difference in the association between non-classic pain and various variables when considering the neck and jaw as classic radiation sites for retrosternal chest pain, compared to when these sites were not included.

A previous study has indicated that men are more likely to report typical anginal pain, characterized by discomfort in the chest and upper arm, whereas women tend to experience atypical angina, manifested as pain in the jaw, neck, shoulders, and back [19]. Another study found that women were more likely to experience atypical symptoms, compared to men [20]. However, our study found that men experienced non-classic/atypical pain more frequently than women, with rates of 64.6% and 52.4%, respectively. This difference, however, was not statistically significant. This discrepancy may be attributed to the lower rates of alcohol consumption and smoking among women in our country, which reduces their risk of developing CAD. This aligns with our study results, which identified a correlation between smoking and non-classic pain.

Another study discovered that 30% of patients did not report chest pain [8], and it was found that chest pain is the dominant and most frequent symptom for both men and women ultimately diagnosed with acute coronary syndrome [6]. While our study indicated that the precordial area was the most frequently reported site of pain, experienced by 88% of the participants. A previous study found that women with MI tend to be older than men with MI. However, our study found no significant age difference between genders for CAD, but did observe that men with CAD tend to be older than women with CAD [20]. A croatian study reported that diabetes mellitus is associated with atypical symptoms, meanwhile our study found no significant association with the type of pain [21]. Specifically, our study revealed that 42.1% of diabetic patients reported experiencing classic pain symptoms.

It is noteworthy that 81.9% of the patients in our study reported experiencing referred pain, which is significant, as many patients tend to overlook this type of pain. Various studies have linked referred pain to the convergence projection theory, which suggests that central neurons receive combined visceral and somatic stimuli, resulting in the perception of both visceral pain and referred somatic pain. Initial research focused on the spinothalamic tract (STT) and the spinoreticular tract (SRT) in the upper thoracic spinal cord due to their role in transmitting somatic pain signals and receiving sensory input from the heart. These pathways were chosen based on their established roles in transmitting somatic pain signals to the thalamus and reticular formation, respectively, and in receiving sensory input from the heart in the upper thoracic cord [19]. Studies in animals demonstrated that stimulating cardiac spinal afferents activated around 80% of STT and SRT cells in the upper thoracic segments T1-T5. Neurons responsible for processing cardiac pain were identified in specific laminae of the spinal gray matter. These neurons exhibited responses to bradykinin applied either epicardially or intracardially to the heart, as well as to coronary artery occlusion. All neurons receiving input from the heart received somatic input, primarily nociceptive signals from the chest and upper limb muscles, which provides support for the convergence projection theory concerning STT and SRT neurons in the upper thoracic cord. They indicate that these neurons may play a role in generating sensations of angina and contributing to the referral of pain to nearby somatic structures [19]. However, 18.1% of the patients reported experiencing localized pain transmitted via spinal cardiac afferent fibers.

In cases of CAD, fissures or erosions in atherosclerotic plaques lead to the release of various chemical mediators such as serotonin, histamine, thromboxane A2, bradykinin, reactive oxygen species including hydroxyl radicals, lactic acid causing proton release, and adenosine which triggers the production of prostaglandins (PGE2 and PGI2) within the coronary artery lumen. These chemical agents, either individually or in combination, interact with specific receptors primarily located on chemically sensitive terminals, resulting in the depolarization of cardiac visceral spinal afferent fibers [19].

Furthermore, our study highlighted that most smokers (72.2%) among our patients presented with non-classic pain, indicating a potential link between smoking and non-classic angina. Previous studies suggested that smokers are at a higher risk to develop back pain and other chronic pain conditions [22, 23]. Another study showed that among patients with chronic pain, smokers complained of higher pain intensity and increased number of pain sites [24, 25]. One underlying mechanism might be that cigarette smoking impairs oxygen delivery to tissues by increasing sympathetic outflow and carboxyhemoglobin levels and causing vasoconstriction. Thus, smoking may accelerate degenerative processes which make the body more vulnerable to injury. This can explain why smoking is a risk factor for osteoporosis, lumber disk diseases and impaired bone healing [26]. This aligns with the results of our study, which found that 25% of smokers had back pain, 26.4% had pain the the right shoulder and 19.4% had pain in the right arm.

In our study, we found that only 38% of patients complained of classic pain, which indicates that the majority of patients suffered from non-classic pain sites. This is a significant result, since atypical symptoms of AMI were associated with less invasive therapy and poor outcome, and in-hospital mortality was significantly higher in atypical than in typical group in a previous study conducted in Japan [27]. Moreover, 12% of patients presented with no precordial pain, whereas a study in Poland [28] showed that only 6.4% presented without chest pain. This is especially important because previously, if the patient did not report chest pain, then they were disadvantaged from even receiving a prehospital ECG [29]. Another important result of our research was that patients with previous heart disease where more likely to have atypical pain, which is consistent with the results of the Japanese study [20]. While in the Polish study [18], previous heart diseases and hypertension were more linked with typical symptoms of MI. It is crucial to highlight all the different symptoms of CAD because these symptoms are the cues for further diagnostic exams such as ECG and cardiac catheterization.

There are a few limitations in this study. The sample size included 100 patients, which may be considered relatively small. However, the extended study period overcomes this limitation by encompassing a wide range of climate changes and conditions, thereby enriching the results. It is worth noting that the study was conducted in a single city, limiting the generalizability of the findings. Nevertheless, Damascus, being the capital of Syria, and the inclusion of hospitals among the largest in the country lend credibility to the study. Another limitation pertains to the potential for patients to exaggerate or misidentify the location of pain. Additionally, as cross-sectional studies do not establish causality, further research is required. As per our findings, it is recommended for healthcare professionals to exercise caution in diagnosing patients and to remain vigilant in recognizing and addressing atypical symptoms, particularly in individuals with known risk factors. Future researchers interested in exploring similar topics are encouraged to conduct studies with larger sample sizes across multiple cities to capture a broader range of variations. Furthermore, investigating the precise relationship between smoking and non-classic pain is also recommended.

## Conclusion

Our study found that non-classic pain is common (62%), occurring mostly in the right shoulder, right arm and the back, and is associated with smoking and previous heart disease. Hopefully, this study will assist doctors in acknowledging the previous risk factors and their contributions to different types of pain, ultimately aiding in the diagnosis of CAD across various pain sites. This study underscores the importance of recognizing all the pain sites that might indicate CAD, especially in smokers who present with non-classical pain sites. Misdiagnosing CAD can have fatal outcomes. Many patients with non-classical CAD symptoms might be deprived of vital pre-hospital procedures, such as ECG, which can be lifesaving.

### Electronic supplementary material

Below is the link to the electronic supplementary material.

Supplementary Material 1

## Data Availability

Data is provided within the supplementary information files.
